# Interleukin 12B (*IL12B*) Genetic Variation and Pulmonary Tuberculosis: A Study of Cohorts from The Gambia, Guinea-Bissau, United States and Argentina

**DOI:** 10.1371/journal.pone.0016656

**Published:** 2011-02-09

**Authors:** Gerard A. J. Morris, Digna R. Velez Edwards, Philip C. Hill, Christian Wejse, Cyrille Bisseye, Rikke Olesen, Todd L. Edwards, John R. Gilbert, Jamie L. Myers, Martin E. Stryjewski, Eduardo Abbate, Rosa Estevan, Carol D. Hamilton, Alessandra Tacconelli, Giuseppe Novelli, Ercole Brunetti, Peter Aaby, Morten Sodemann, Lars Østergaard, Richard Adegbola, Scott M. Williams, William K. Scott, Giorgio Sirugo

**Affiliations:** 1 MRC Laboratories, Fajara, The Gambia (West Africa); 2 Unità di Genetica Medica, Ospedale San Pietro FBF, Rome, Italy; 3 Dr. John T. Macdonald Foundation Department of Human Genetics and Hussman Institute of Human Genomics, University of Miami, Florida, United States of America; 4 Centre for International Health, University of Otago School of Medicine, New Zealand; 5 Department of Infectious Diseases, Aarhus University Hospital, Skejby, Denmark; 6 Bandim Health Project, Danish Epidemiology Science Centre and Statens Serum Institute, Bissau, Guinea-Bissau; 7 Centro de Educación Médica e Investigaciones Clínicas “Norberto Quirno” (CEMIC), Division of Infectious Diseases, Department of Medicine, Buenos Aires, Argentina; 8 Hospital F.J. Muñiz, Department of Medicine, Buenos Aires, Argentina; 9 Family Health International, Research Triangle Park, North Carolina, United States of America and Duke University Medical Center, Durham, North Carolina, United States of America; 10 Dipartimento di Biopatologia e Diagnostica per Immagini, Università di Tor Vergata, Rome, Italy; 11 University of Southern Denmark, Odense, Denmark; 12 Center for Human Genetics Research, Vanderbilt University, Nashville, Tennessee, United States of America; Fundació Institut Germans Trias i Pujol; Universitat Autònoma de Barcelona CibeRES, Spain

## Abstract

We examined whether polymorphisms in interleukin-12B (*IL12B*) associate with susceptibility to pulmonary tuberculosis (PTB) in two West African populations (from The Gambia and Guinea-Bissau) and in two independent populations from North and South America. Nine polymorphisms (seven SNPs, one insertion/deletion, one microsatellite) were analyzed in 321 PTB cases and 346 controls from Guinea-Bissau and 280 PTB cases and 286 controls from The Gambia. For replication we studied 281 case and 179 control African-American samples and 221 cases and 144 controls of European ancestry from the US and Argentina. First-stage single locus analyses revealed signals of association at *IL12B* 3′ UTR SNP rs3212227 (unadjusted allelic p = 0.04; additive genotypic p = 0.05, OR = 0.78, 95% CI [0.61–0.99]) in Guinea-Bissau and rs11574790 (unadjusted allelic p = 0.05; additive genotypic p = 0.05, OR = 0.76, 95% CI [0.58–1.00]) in The Gambia. Association of rs3212227 was then replicated in African-Americans (rs3212227 allelic p = 0.002; additive genotypic p = 0.05, OR = 0.78, 95% CI [0.61–1.00]); most importantly, in the African-American cohort, multiple significant signals of association (seven of the nine polymorphisms tested) were detected throughout the gene. These data suggest that genetic variation in *IL12B*, a highly relevant candidate gene, is a risk factor for PTB in populations of African ancestry, although further studies will be required to confirm this association and identify the precise mechanism underlying it.

## Introduction

Tuberculosis (TB) contributes significantly to global morbidity and mortality, with an estimated 2 billion individuals infected worldwide (http://www.who.int/mediacentre/factsheets/fs104/en/). TB will likely represent an increasing social and economic burden, particularly in resource-poor countries, because of newly emerging multi-drug resistant strains of *Mycobacterium tuberculosis* (*Mtb*). Over the next 20 years the World Health Organization (WHO) estimates that one billion people will become newly infected and more than 25 million will die from TB, despite the availability of antibiotics [1]. The impact of the continuing TB epidemic will likely be greatest in sub-Saharan Africa, with incidence rates ranging from 50 to greater than 300 per 100 000 individuals [2]. In 2005 there were approximately 3.8 million African TB cases and 550 000 TB-caused deaths with more than 2.5 million of the cases being new and accounting for 29% of the worldwide incidence (http://www.who.int/mediacentre/factsheets/fs104/en/). In Africa the study of TB is complicated by the parallel epidemic of HIV because co-morbidity is common, making it necessary to consider HIV infection, especially in high HIV prevalence areas.

The vast majorities of those infected with Mtb maintain the bacterium in a latent state, and fail to develop overt clinical disease; however, they remain at risk of progressing to disease later in their lives. The factors determining risk of infection and progression to active disease are multifactorial, involving host–pathogen interactions and environmental components [3]. In addition, innate and adaptive immunity are critical to the elimination or containment of Mtb, with macrophages playing a key role [4]. Central to the pathogenesis of TB therefore, is the inability of infected macrophages to contain Mtb and the failure of T cells to confer long-lasting protective immunity [2]. In some this may be due to genetic predispositions, and functional single-nucleotide polymorphisms (SNPs) in key genes have been associated with susceptibility and/or resistance to TB [5]–[7]. In particular, functional/putatively functional SNPs in genes that affect macrophage anti-mycobacterial activity (*e.g*., vitamin D receptor [*VDR*], monocyte chemoattractant protein [*MCP–1*], and interferon [*IFNγ*]) associate with increased risk for and severity of tuberculosis in various ethnic groups [6]–[9].

Compelling evidence indicates that different T cell populations, including CD4+, CD8+, double-negative, and γδ T cells, participate in protective immunity against TB [10], at least in part due to their ability to secrete IFNγ. A series of Mendelian immune disorders of the IL12/IFNγ axis have been described and all markedly predispose individuals to severe mycobacterial diseases [11]–[15]. Interleukin-12 (IL12) is a heterodimeric cytokine composed of two subunits, p35 and p40, which are encoded by the *IL12A* and *IL12B* genes, respectively. *IL12B* gene expression is a major inducer of IFNγ and IL12-p40 is required for IFNγ-induced protection from Mtb. The IL12-p40 subunit also increases production of IFNγ by covalently linking to the p19 subunit of yet another cytokine, IL23. This linkage can promote IFNγ response in absence of IL12-p35 [13], [16]. Additionally, *IL12B* expression is significantly decreased in patients with active TB compared to individuals with latent TB infection, consistent with the hypothesis that low expression of IFNγ in TB patients is due to the reduction of IL12-p40 [17]. Addition of exogenous IL12 enhanced the IFNγ response of ESAT-6-stimulated peripheral blood mononuclear cells (PBMC) from TB patients, underscoring the importance of IL12B in the induction of IFNγ [17]. Thus the IL12 role in TB is of increasing interest as rare *IL12B* gene mutations cause altered IL12-p40 levels and increased susceptibility to mycobacterial infection (including non-tuberculous mycobacteria) because of the impaired IFNγ mediated immunity [11], [13]–[15], [18].

The purpose of this study was to examine whether polymorphisms in the *IL12B* gene were associated with susceptibility to pulmonary tuberculosis in two West African populations – from The Gambia and Guinea-Bissau – and to replicate these findings in independent samples from North and South America. The incidence of PTB in Guinea-Bissau is among the highest in the world (470/100 000). The study area has a population of 92 000 and is composed of several ethnic groups including Papel (32%), Manjaco (14%), Mancanha (10%), Balanta (9%), Fulani (13%), Mandinka (7%) and others (15%). The incidence of PTB in the Gambia is 258/100 000. More than 90% of the population of The Gambia is from five main ethnic groups, including Mandinka, Fulani, Wolof, Jola/Karoninka, and Sarahule (a general demographic description of The Gambia can be found at http://www.columbia.edu/~msj42/index.htm). In total nine polymorphisms were examined in 321 PTB cases and 346 controls from Guinea-Bissau and 280 PTB cases and 286 controls from The Gambia (however, due to limited availability of DNA, the microsatellite and the in/del were typed in a slightly reduced number of samples from The Gambia, see Materials and Methods). Results were then examined in a mixed case-control replication dataset consisting of PTB cases, relatives, or close contact controls from 281 cases and 179 controls African-Americans and 221 cases and 144 controls Caucasians.

## Results

Clinical and demographic information for Guinea-Bissau, The Gambia, and the African-American and Caucasian cohorts has been previously described [19]–[21]. Examination of allele and genotype frequency differences for cases with and without HIV indicated no evidence for significant differences between the two groups in any of the populations examined; as a result analyses were performed pooling cases with and without HIV. A list of the polymorphisms examined, their base-pair positions, and their potential role in the gene are provided on Table 1. Differentiation among ethnic groups (ethnicity was self-identified) within cases and controls, as measured by F_st_ were very small in Guinea-Bissau and The Gambia (less than |0.01| in all cases), indicating little to no differentiation among ethnic groups within cases and controls for any of the polymorphisms examined (Table 1). Tests for allele and genotype frequency differences between Guinea-Bissau and The Gambia cohorts showed only one significant difference between the two cohorts: SNP rs3212227 differed in both allele (p<1.00×10^−3^) and genotype (p = 1.00×10^−6^) comparisons in cases only (Table S1).

**Table 1:** F_st_ information by locus.

*Gene*	Marker	Position	Role	Guinea-Bissau		The Gambia	
				F_st_ a		F_st_ a	
				Cases	Controls	Cases	Controls
*IL12B*(5q33.3)	rs3212227	158675528	3′ UTR	0.0014	−0.0046	0.0004	−0.0016
	rs11574790	158676424	Intron 6	0.0032	−0.0066	−0.0051	−0.0011
	rs2421047	158678885	Intron 5	0.0022	−0.0083	−0.0045	−0.0065
	rs919766	158680142	Intron 4	−0.0013	−0.0043	−0.0012	0.0253
	rs2288831	158682591	Intron 3	−0.0019	−0.0063	0.0003	−0.0067
	rs10631390	158683690	Intron 2	0.0021	0.0013	−0.0028	−0.0093
	rs3212220	158686773	Intron 1	0.0003	0.0018	−0.0040	−0.0058
	rs6894567	158689546	Intron 1	−0.0041	−0.0058	−0.0022	0.0094
	rs17860508	158692783	Promoter	0.0010	0.0029	−0.0037	0.0064

a F_st_ was calculated as θ_P_ within each country and within cases and controls. Unlike F_st,_ θ_P_ can be negative.

### Guinea-Bissau

Single locus tests of association identified one significant single locus effect (Table 2a) in *IL12B* at SNP rs3212227 for the allelic test of association (p = 0.04). The best genotypic model for *IL12B* rs3212227 was additive with an OR = 0.78 [95% CI 0.61–0.99] (p = 0.05) (Table 3). When we corrected the genotypic association for gender, ethnicity and age as covariates neither the p value changed (0.040 for the additive model vs. 0.044 for the adjusted model) nor the effect size changed noticeably (0.78 for the additive model unadjusted vs. 0.77 for the adjusted model) (Table S2). There were no significant deviations from Hardy Weinberg Equilibrium (HWE) in cases or controls.

**Table 2:** Single locus allelic and genotypic tests of association in Africans (2a), African-Americans (2b), and Caucasians (2b).

a	*Gene*	Marker	MinorAllele	Control MAF		Case MAF		*P*-Value			
				GuineaBissau	TheGambia	GuineaBissau	TheGambia	Guinea-Bissau		The Gambia	
								Allele	Genotype	Allele	Genotype
	*IL12B*	rs3212227	G	0.31	0.36	0.26	0.40	0.04	0.13	0.19	0.43
		rs11574790	A	0.25	0.27	0.24	0.22	0.67	0.88	0.05	0.15
		rs2421047	A	0.34	0.34	0.35	0.38	0.76	0.94	0.14	0.24
		rs919766	C	0.27	0.29	0.26	0.28	0.90	0.84	0.78	0.95
		rs2288831	C	0.39	0.35	0.38	0.38	0.64	0.75	0.29	0.61
		rs10631390	B^a^	0.36	0.34	0.38	0.37	0.54	0.34	0.36	0.63
		rs3212220	A	0.37	0.37	0.37	0.38	0.71	0.58	0.48	0.77
		rs6894567	G	0.34	0.31	0.35	0.35	0.84	0.55	0.22	0.42
		rs17860508	D^a^	0.31	0.29	0.33	0.32	0.45	0.74	0.35	0.68

b	*Gene*	Marker	MinorAllele	Control MAF		Case MAF		*P*-Value			
				GuineaBissau	TheGambia	GuineaBissau	TheGambia	Guinea-Bissau		The Gambia	
								Allele	Genotype b	Allele	Genotype b
	*IL12B*	rs3212227	G	0.31	0.36	0.26	0.40	0.04	0.13	0.19	0.43
		rs11574790	A	0.25	0.27	0.24	0.22	0.67	0.88	0.05	0.15
		rs2421047	A	0.34	0.34	0.35	0.38	0.76	0.94	0.14	0.24
		rs919766	C	0.27	0.29	0.26	0.28	0.90	0.84	0.78	0.95
		rs2288831	C	0.39	0.35	0.38	0.38	0.64	0.75	0.29	0.61
		rs10631390	B^a^	0.36	0.34	0.38	0.37	0.54	0.34	0.36	0.63
		rs3212220	A	0.37	0.37	0.37	0.38	0.71	0.58	0.48	0.77
		rs6894567	G	0.34	0.31	0.35	0.35	0.84	0.55	0.22	0.42
		rs17860508	D^a^	0.31	0.29	0.33	0.32	0.45	0.74	0.35	0.68

*In bold are statistically significant (p≤0.05).

a rs10631390 is a microsatellite and was coded so that alleles 131 and 134 were grouped into one category coded B here and the other alleles 125 and 128 were grouped into another category coded A. rs17860508 is an insertion deletion and the deletion (D) was considered the minor allele.

b Genotype p-values for African-American and Caucasian samples are additive genotype models performed using GEE.

**Table 3:** OR for significant replicated associations from Table 2.

Populations	Model (rs3212227)	OR	95% CI	*P*-Value
Guinea-Bissau	Additive (TT (major allele homozygote), GT, GG (minor allele homozygote))	0.78	0.61–0.99	0.05
	TT&GT v GG (Baseline)	1.65	0.91–3.05	0.08
	GG&GT v TT (Baseline)	0.77	0.56–1.07	0.11
The Gambia	Additive (TT (major allele homozygote), GT, GG (minor allele homozygote))	1.18	0.91–1.55	0.21
	TT&GT v GG (Baseline)	0.8	0.45–1.39	0.39
	GG&GT v TT (Baseline)	1.24	0.84–1.84	0.25
African-American	Additive (TT (major allele homozygote), GT, GG (minor allele homozygote))	0.78	0.61–1.00	0.05
	TT&GT v GG (Baseline)	1.21	0.72–2.03	0.48
	GG&GT v TT (Baseline)	0.69	0.49–0.98	0.04
Caucasian	Additive (TT (major allele homozygote), GT, GG (minor allele homozygote))	0.89	0.58–1.37	0.59
	TT&GT v GG (Baseline)	0.96	0.22–4.20	0.95
	GG&GT v TT (Baseline)	0.85	0.51–1.42	0.53

*In bold are statistically significant (p≤0.05).

**Figure 1:**
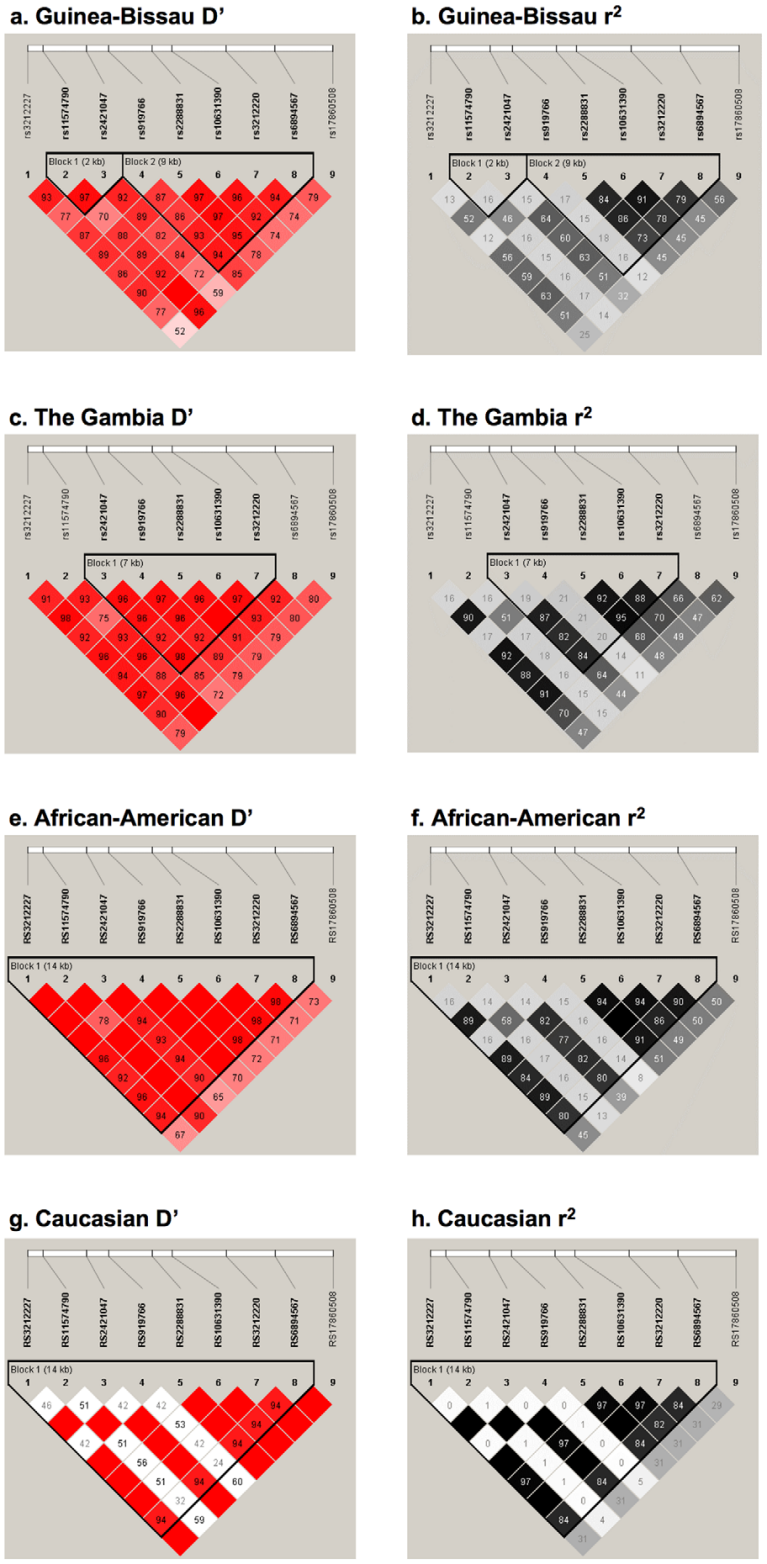
Haploview *IL12B* linkage disequilibrium plots for controls. All figures are oriented 5′ to 3′, right to left, relative to the gene orientation on the minus strand. Linkage disequilibrium (LD) plots characterizing haplotype blocks in *IL12B* in Guinea-Bissau (Figure 1a and 1b), The Gambia (Figure 1c and 1d), African-Americans (Figure 1e and 1f), and Caucasians (Figure 1g and 1h). In the first column are LD plots for pairwise D' between markers and in the second column are LD plots for pairwise r^2^ between markers. Both r^2^ and D' values are indicated in percentages within squares in the LD plot. Strong LD is indicated by dark gray/red, while light gray/pink and white indicate uninformative and low confidence values, respectively. LD Blocks were created with the default algorithm in HaploView program (version 4.1) that creates 95% confidence bounds on D' considered being in strong LD where 95% of the comparisons made are informative.

Examination of the linkage disequilibrium (LD) structure of *IL12B* in controls (Figure 1a and 1b) revealed strong overall D' with D' ranging between 0.86–0.97 in the 5′ block. An evaluation of r^2^ indicates that the 5′ region (rs6894567 to rs2288831) is separate from the 3′ region of the gene. Of note this overall pattern of LD is very similar to that in the cases (Figure S1, a and b).

### The Gambia

Single locus tests of association in The Gambia identified a significant allelic association at *IL12B* SNP rs11574790 (Table 2a) (p = 0.05). The most significant genotype effect for this SNP was detected for an unadjusted additive model with an OR = 0.76 [95% CI 0.58–1.00] (p = 0.05). Adjusting for gender, ethnicity and age only slightly changed the p value to 0.069 and the OR to 0.78 [95% CI 0.58–1.02]. There were no statistically significant deviations from HWE in cases or controls.

LD of *IL12B* in The Gambian controls (Figure 1c and 1d), shows that D' of rs11574790 with the SNP associated in the Guinea-Bissau population (rs3212227) is large (0.91, Figure 1c). The r^2^ between these two SNPs (Figure 1d), however, was marginal (r^2^ = 0.16), but slightly stronger than in Guinea-Bissau (0.13). Again it is important to note that the overall LD structure is similar in the cases from The Gambia (Figure S1, c and d).

### African-Americans and Caucasians

There were no statistically significant deviations from HWE in African-American or Caucasian cases or controls. Single locus tests of association in the African-Americans identified seven significant allelic associations at *IL12B* polymorphisms rs3212227 (p = 0.002), rs2421047 (p = 0.008), rs2288831 (p = 0.008), rs10631390 (p = 0.001), rs3212220 (p = 0.003), rs6894567 (p = 0.007), and rs17860508 (p = 0.002) and two significant genotypic associations at rs3212227 (p = 0.05) and rs6894567 (p = 0.03) (Table 2b). These data replicated the association observed in Guinea-Bissau at rs3212227, with an OR = 0.78 (95% CI [0.61–1.00, p = 0.047]) (Table S3a). The lack of association in the Caucasian sample may be due to the unusual reduction in LD in the Caucasian samples relative to the ones of African ancestry (Figure 1g and 1h).

There appears to be stronger LD and larger LD block structure for *IL12B* in African-Americans controls (Figure 1e and 1f) relative to controls from Guinea-Bissau (Figure 1a and 1b), The Gambia (Figure 1c and 1d), and in Caucasians (Figure 1g and 1h), with one large D' LD block identified relative to the two blocks identified in both The Gambia and Guinea-Bissau. This may represent an effect of admixture causing more extensive LD; this LD structure and the larger number of single polymorphism associations provide evidence of association with PTB. As noted above case LD structures are consistent with controls from the same sample (Figure S1).

## Discussion

In this study we observed significant or marginally significant associations of PTB with *IL12B* intragenic variants in three independent populations: The Gambia, Guinea-Bissau and African-Americans. Although the results in terms of the exact SNP showing association differed between the two West African populations, and despite small physical distance between the two associated polymorphisms (3′UTR rs3212227 in Guinea-Bissau and rs11574790 in intron 6 in The Gambia), this is not unexpected given the noticeable differences in LD structure and the differences in haplotype frequencies between Guinea-Bissau and The Gambia (data not shown). In African-Americans an association was detected with rs3212227 (thus replicating and reinforcing the finding in the Bissau cohort), however, in this sample, allelic association signals were detected throughout the *IL12B* gene, from the 3′ end to rs17860508, the 7-bp deletion and 3-bp insertion in the promoter. The association pattern differs in African-Americans with respect to West Africans (i.e. broad vs. localized signal) and this can be explained by the lower levels of LD observed in the Gambian and Bissau samples (Figure 1), translating into a lower chance of detecting association over extended genomic distances in these populations. In addition, it is important that the direction of association is the same in all three populations, further reinforcing a conclusion of putative association. However, we recognize that adding covariates to the genotype model impacted some, but not all of the associations. For example, the p value did lose significance for rs11574790 in The Gambian samples, but the effect size remained very similar and the reduction in significance is likely due to a reduction in power when additional variables are added to the statistical model. Therefore, we caution that the evidence of a common effect across populations of African ancestry, although reinforcing a possible association, will require follow-up and replication to confirm them.

One potential caveat with the African-American result lie in the fact that this population is admixed with Europeans, and that admixture levels can be variable. Therefore, *IL12B* allele frequencies could differ between cases and controls as a reflection of population stratification. Although we acknowledge that the issue could have been addressed by typing ancestry-informative markers, these were not available for this study. In support of lack of population stratification artefacts is that controls were drawn from the same residential area of cases and therefore we would expect that admixture in the two groups is similar. Additionally, the lack of deviation from HWE and the consistency of LD patterns between cases and controls indicate that stratification is limited, if it exists at all. Lastly, the minor allele frequencies across the populations of African descent for SNPs that show association differ by less than or equal to only 7% (Table 2), indicating that stratification is not a major factor.

It is likely that the *IL12B* associations reported here are in fact due to an unknown variant(s) that is in LD with the polymorphism positively associated with TB in this study; the discrepancy between population samples could simply be due to distinct patterns of genetic variation that is not fully explained by the markers we assayed. Recent studies would support this point as there are strikingly different genetic backgrounds across the African continent, especially compared to non-African populations, despite substantial shared ancestry [22]. This is further supported by the differences between both the Guinea-Bissau and The Gambia samples as compared to the data from the Yoruba samples from the HapMap project.

### Yoruba

*IL12B* haplotype frequencies (parental haplotypes only) showed significant differences in haplotype frequencies compared to controls from Guinea-Bissau and The Gambia. There was also stronger overall LD in the Yoruba (Figure S2, b and d). We also observed stronger associations in *IL12B* in African-Americans with similar effect sizes to Guinea-Bissau, indicating that we are perhaps seeing more associations in African-Americans due to stronger overall LD, possibly due to the relatively recent admixture that formed this population. These comparisons confirm that “neighboring” African populations might display considerable variability in LD, and suggests that functional variants at the 3′ end of *IL12B* may be differentially tagged in these West African populations. Hence, association of nearby polymorphisms (e.g., rs3212227 and rs11574790) can be reasonably interpreted as strong evidence for association with *IL12B*. Proof of this will require additional fine mapping in order to better understand the role of *IL12B* in PTB. Unfortunately, haplotype analyses did not refine a smaller region of association as compared to that detected by single SNPs nor did haplotype analyses improve significance levels (data not shown); rather haplotypes turned out to be uninformative due to the larger number of haplotypic variants generated by the polymorphisms (including a microsatellite) typed across the gene.

Our findings are interesting because of the consistency we were able to demonstrate in these distinct populations, yet there are some potential limitations to be considered. First, TB disease was defined somewhat differently in the original populations from The Gambia and Guinea-Bissau, compared with disease definition in the replication dataset since none of the former samples were culture-confirmed, while all of the latter were culture-confirmed. This potential heterogeneity in case definition is less concerning because, if anything, it is more likely to reduce the chances of finding real differences between cases and controls. Similarly, controls were selected from households in Guinea-Bissau where there was no evidence of recent TB exposure, whereas the other sites included and even sought controls that had been exposed to the index case. In addition, we had HIV data on 70% of the American samples, 90% of the Guinea-Bissau samples and 87% of The Gambia. We included the small number of known HIV-positive cases in our analyses, which could result in confounding by ascertainment center or HIV status. To account for these effects we adjusted for ascertainment site in our models and conducted a sensitivity analysis of HIV status by removing HIV-positive cases from our analysis. However, HIV status did not influence the significance of our results and was therefore not reported explicitly.

We observed some baseline differences for the ratio of males to females and mean age at examination in cases and controls. We adjusted for these effects in our models, but these variables do not affect our point estimates of the effect size substantially, with changes in OR much less than 0.10, indicating that these variables are not confounders. We did not correct for multiple testing in our analyses, but our study design was to replicate any association signal detected in West Africans in subsequent cohorts, i.e., the African-American and the Caucasian, an approach that had the advantage of minimizing false positive results. The fact that SNP rs3212227 was replicated and had a small allelic p value in the African-Americans (p = 0.002) suggests that this design did in fact enable us to identify a real association, worthy of follow-up in future studies.

The results of this study implicate *IL12B* as a susceptibility gene for PTB in West Africans from The Gambia and Guinea-Bissau and in African-Americans. Although negative associations between *IL12B* and PTB have been reported in small (underpowered) population-based studies [23], [24], our results, indicating a role for *IL12B* variation in TB susceptibility, are in agreement with studies in Hong Kong Chinese [25], Russian [16], Indian [26] and Indonesian population samples [27]. In addition a recent study from South Africa, that analyzed an admixed cohort from Cape Town, showed weak evidence for association with *IL2B* variation, including some of the polymorphisms we examined [28]. Taken together, our multi-ethnic replication study suggests that *IL12B* sequence variation influences risk for pulmonary PTB either directly or through LD with a functional variant. Of all the polymorphisms we tested, only rs17860508 (insertion/deletion) and SNP rs3212227 (corresponding to a TaqI restriction site) have been shown or suggested to have functional effects: rs17860508 on *IL12B* mRNA levels [29] and rs3212227 on IL12p70 secretion [30]. The fact that not all studies find a clear association, does not by itself invalidate the several positive findings. It has been shown that in an epistatic 2 locus model, the effects of a single gene may be impacted by variants elsewhere in the genome and that when variants at a second unmeasured locus differ in allele frequencies among populations power to detect association at the first locus can be changed substantially among them [31]. It is noteworthy that rs3212227, which was replicated in the African-American cohort lies in an evolutionarily conserved region (analyses not shown; Vista Browser 2.0, http://genome.lbl.gov/vista/index.shtml), reinforcing the idea that the 3′ end of gene might play a significant functional role *in vivo* [32]. Elucidation of the role of *IL12B* in susceptibility to PTB requires isolating the functional variants in *IL12B* most affecting the pathogenesis of disease. In addition, isolation of *IL12B* variants related to pathogenesis of PTB could result in the identification of susceptible individuals as well as in a better understanding of factors modulating vaccine-dependant responses for which *IL12* promotes and sustains Th1 immunological memory [33].

## Materials and Methods

### Study Populations

#### Guinea-Bissau

This case-control study was conducted at the Bandim Health Project (BHP), a demographic surveillance site in Bissau, the capital of Guinea-Bissau. BHP has followed this population since 1978. The ethnic breakdown of these data from Guinea-Bissau sample have been previously reported [19]. Patients included in the study (cases) were residents or long-term guests of Bissau, aged >15 years, newly PTB-diagnosed using three sputum examinations for acid fast bacteria or clinical criteria by the World Health Organization's definition of active pulmonary PTB [34]. No culture confirmation of TB was available in Bissau, during the study period, as facilities were destroyed during a civil war; 218/321 (68%) were smear positive. Patients with newly diagnosed TB were enrolled when they started antitubercular treatment at local health centers. During the inclusion period from November 2003 to November 2005, 438 TB patients were screened at local health centers: 344 subjects met inclusion criteria and accepted participation, and from these we could obtain 321 DNA samples. There were no exclusion criteria.

Healthy controls were recruited from the study area from May 2005 to November 2005. A random sample of 200 houses was selected from the database of all subjects living in the study area, and houses with a recorded case of TB within the past 2 years were excluded from the sampling. Exclusion criteria for controls included the presence of cough for more than 2 weeks, history of TB and TB in the household within the last 2 years to avoid households with a high-risk of active TB. The composition of the case and control samples was different in terms of sex and ethnicity. These differences are due to the sampling strategy as controls were derived from healthy nuclear families; hence more healthy married couples were collected, whereas TB patients are more often males. The ethnic differences are due to willingness of healthy subjects to give blood, which was not the same across the ethnic groups, whereas most TB patients agreed to participate in the study regardless of their ethnic background.

All subjects were interviewed by field assistants, using a standardized questionnaire on ethnicity, environmental factors and prior exposure to TB. Permission to perform HIV tests was asked for cases but not for controls, as requiring HIV testing would have negatively impacted participation in the study. Venous blood samples (4 ml in ethylenediaminetetraacetic acid) were collected from all subjects. Ethical approval was granted by the ‘Unidade de Coordenacao de Estudos e Pesquisas em material de Saude’ (Ministry of Health) in Guinea-Bissau. All adults and children's guardians signed an informed consent to the study.

#### The Gambia

 Between June 2002 and October 2004, PTB cases and their household contacts were enrolled in a prospective cohort study in the Greater Banjul region of The Gambia, where about 750 000 people live, i.e. more than 50% of the total Gambian population (http://www.columbia.edu/~msj42/index.htm). Our Gambian cohort consisted of Mandinka (34%), Jola (29%), Wolof (13%), Fulani (9%), Serere (6%), Manjaco (3.5%), Sarahule (1%), Aku (1.%) and others (3.5%). According to WHO 2007 burden estimates, the incidence of TB in The Gambia is 258/100 000 and 11% of new TB cases are HIV positive (http://www.who.int/globalatlas/predefinedReports/TB/PDF_Files/gmb.pdf). Recruitment took place from the major government TB clinic and the Medical Research Council (MRC) outpatients' clinic, and consisted of sputum smear positive pulmonary TB cases aged at least 15 years old, who had at least one household contact living with them [20]. Included patients (screened for HIV) had two sputum smear samples positive for acid-fast bacilli and with *M. tuberculosis* isolated upon culture. An index case was defined as the first TB case identified in a household. Household contact controls were defined as individuals at least 6 months of age living the majority of the time on the same compound as the respective index TB case, sharing meals and identifying a common household head. Written informed consent was obtained from all subjects. The study was approved by the combined Gambia Government/MRC National Ethics Committee of The Gambia.

As with Guinea-Bissau subjects were interviewed by field assistants, using a standardized questionnaire on ethnicity, environmental factors and prior exposure to TB. Permission to perform HIV tests was asked for cases but not for controls. Venous blood samples (4 ml in ethylenediaminetetraacetic acid) were collected from all subjects, and from these, 286 control DNA samples and 280 case DNA samples were genotyped for analyses. All samples were archived in the National Gambian DNA Bank and used in compliance with the bank guideline [35].

#### African-Americans and Caucasians

Participants were ascertained through the North Carolina or South Carolina TB Control Programs, U.S.A., or as patients at the outpatient clinic at F.J. Muñiz Hospital in Buenos Aires, Argentina, between 2002 and 2006. Criteria for inclusion as TB cases were: a) age 14 years or older and culture-confirmed pulmonary TB, or b) younger than 14 years old and either culture-confirmed or clinically diagnosed pulmonary TB that included a positive tuberculin skin test plus an infiltrate or hilar adenopathy on chest x-ray. Individuals were eligible to participate if TB had been diagnosed in the past, or if they were currently receiving TB treatment. Family members of eligible TB cases, who themselves had a history of TB, were enrolled as part of a multi-case family if review of their records established diagnosis of pulmonary TB. Details on the study population are provided by Velez et al. [21].

Severity of TB disease was assessed by presence of acid-fast bacilli (AFB) in sputum smears or x-ray evidence of cavitary lesions. We attempted to document HIV status through medical record review for all subjects. However, participation in this study did not require that the individual authorize review of HIV test results.

Unaffected individuals who were in close contact with cases (household contacts such as spouses and partners, and relatives such as parents and siblings) were enrolled as controls.

Informed consent was obtained from all subjects or their legal representatives before participation in the study. Human experimentation guidelines of the U.S. Department of Health and Human Services and those of the participating research institutions were followed. The protocol was IRB-approved at Duke University Medical Center, the North and South Carolina Departments of Public Health (USA), Centro de Educación Médica e Investigaciones Clínicas “Norberto Quirno” (CEMIC), the F.J. Muñiz Hospital, Buenos Aires, Argentina, and the University of Miami Miller School of Medicine.

### DNA extraction and genotyping

All DNA samples were extracted using a standard salting-out procedure (Guinea-Bissau and The Gambia) or the Puregene method from Gentra systems (African-Americans and Caucasians). DNA purities were estimated spectrophotometrically, and final concentrations were determined by PicoGreen. For Guinea-Bissau and The Gambia samples (4 ng of DNA) were genotyped by TaqMan assays (ABI, Applera International Inc, Foster City, CA, USA) in 10 µl reaction volume, using the Rotor-Gene 3000 (Corbett Robotics Pty Ltd, Brisbane, Queensland, Australia) and the ABI 7500 real-time PCR systems. Fluorescence curves were analyzed with the Rotor-Gene Software version 6 and the 7500 Sequence Detection Software version 1.2.1 for allelic discrimination. Replication genotyping in African-American and Caucasian samples also used TaqMan assays on an ABI 7900 instrument (ABI, Applera International Inc, Foster City, CA, USA). All samples were analyzed for seven SNPs, one in/del and one microsatellite that were selected based on physical location, minor allele frequency 0.20 in the Yoruba population of the HapMap dataset (http://www.hapmap.org), assay availability and/or a previously reported association. Of these nine variants one SNP in *IL12B* was in a 3′UTR region (rs3212227); one polymorphism was in the *IL12B* promoter region (in/del rs17860508; typed according to [25]); the remaining SNPs were in intronic regions; one polymorphism was a microsatellite (rs10631390, previously known as marker *D5S2941*) in intron 2 [36], [37], typed according to the method described by [37] with a slight modification in primers design: 5′FAM-TCACCAGTGGAGATTTTCATTC3′ (forward) and 5′ TTGGCCTCAGTACGCTTCTT 3′ (reverse) were used for genotyping (Table 1). The genotyping of West African samples was completed in two separate experiments. For the first experiment seven markers were genotyped in 321 PTB cases and 346 controls from Guinea-Bissau and in 280 PTB cases and 286 controls from The Gambia. In the second experiment two markers (rs10631390 and rs17860508) were typed in the 321 PTB cases and 346 controls from Guinea Bissau and in 231 PTB cases and 251 controls in The Gambia. Therefore, due to a limited availability of DNA, only 482 Gambian samples were genotyped in the second experiment, all of which however overlapped with the set of 566 analyzed in the first experiment.

### Bioinformatics Tools

SNP positions (base pair [bp]) and function were identified using the SNPper (http://snpper.chip.org) database NCBI Build 35.1 (Table 1). The HapMap database (http://www.hapmap.org) was used to obtain linkage disequilibrium (LD) and genotype information from the Yoruba and CEPH populations.

### Statistical Methods

#### Quality control

All analyses were performed separately for the four cohorts (Guinea Bissau, The Gambia, African-Americans, and Caucasians). Because the samples from Guinea Bissau and The Gambia were known to contain individuals from different ethnic groups we wanted to test if the polymorphisms we assayed showed evidence of differences among groups. We used Wright's inbreeding coefficient F_st_ to assess this, the F_st_ statistic tests for reduction in heterozygosity due to population subdivision [38]. The rule of thumb proposed by Wright is that values of F_st_ less than 0.05 indicate little or no differentiation among populations [39]. We used Tools for Population Genetics Analysis statistical software, version 1.3 (available at http://www.marksgeneticsoftware.net/) to measure F_st_ [40]. For Guinea Bissau and The Gambia we also performed direct comparisons of allele and genotype frequencies for both cases and controls as well as for common ethnic groups between the two populations, tests for deviations from Hardy Weinberg Equilibrium (HWE), and tests for single locus allele and genotype frequency differences using Powermarker statistical software [41], [42]. Tests for deviations from HWE in African-Americans and Caucasians were calculated using genetic data analysis (GDA) software [43]. Statistical significance for these analyses was determined using Fisher's exact test.

Pairwise LD was characterized and haplotype frequencies were calculated using HaploView [44] statistical software. Standard summary statistics D' and r^2^ were calculated using HaploView [45]. D' is a normalized measure of association among SNPs in a population [46] and r^2^ is the correlation of SNPs in a population that takes into account differences in allele frequencies and is less sensitive to inflation due to small sample size; both are measures of the patterns of allelic association within a population sample, with values 0 and 1 corresponding to no and maximum possible LD, respectively. Haplotype blocks were assigned, using the D' confidence interval algorithm created by Gabriel et al. [47].

#### Single polymorphism association

 Single locus tests of allelic and genotypic association in Guinea-Bissau and The Gambia were performed using a Fisher's exact test available in Powermarker software and followed-up with logistic regression for measures of odds ratios (OR) and confidence intervals (CI). Logistic regression was used modeling additive (0 (homozygous major allele) vs. 1 (heterozygous) vs. 2 (homozygous minor allele)), dominant (0 (homozygous major allele) vs. 1 (heterozygous and homozygous minor allele)), and recessive (0 (homozygous major allele) vs. 1 (homozygous minor allele)) genotypic models. All logistic regression models were further examined with adjustments for potential confounders, ethnicity and gender, for potential inclusion in the regression model presented in the manuscript. Criteria of a change in effect size greater than or equal to 0.10 was determined to indicate evidence for statistical confounding. Neither gender nor ethnicity showed significant evidence for confounding effects and are therefore not included in the regression models presented in the main text. Nonetheless, analyses were still run adjusting for these parameters because stratification may play a role in association (see Table S2). Logistic regression analyses were performed using STATA 9.0 statistical software, College Station, TX.

For African-Americans and Caucasians single locus genotypic tests of association (additive model) were performed with generalized estimating equations (GEE) using the independence correlation matrix implemented in SAS (PROC GENMOD) statistical software version 9.1 (SAS Institute, Cary, NC). GEE performs case-control comparisons in family data by accounting for correlations among related individuals such as concordant and discordant siblings, relative pairs, as well as including unrelated close contacts. GEE performs a valid test of gene x gene and gene x environment interactions in mixed family and case-control data [48]. We also performed GEE analyses adjusting for the potential confounders, age and gender for all analyses. The point estimates did not significantly change (change in OR <0.10), although p values did, when including age and gender for all analyses (see Table S3), supporting the conclusion that for these analyses age and gender are not confounders. Family-based tests of allelic association were performed with Association in the Presence of Linkage (APL) test [49]. APL provides a test of association in the presence of linkage that also estimates missing parental genotypes in nuclear families by using identity by descent (IBD) parameters and pedigree information. However, APL does not permit adjustment for covariates so allelic analyses do not include these adjustments. For Caucasians we also incorporated ascertainment site in all models in order to account for potential genetic heterogeneity between ascertainment sites. GEE and APL were used for analyses in African-Americans and Caucasians due to the different data structures of the populations being examined (e.g. African-Americans and Caucasians consist of related individuals while the West African populations are unrelated cases and controls).

Supplementary information is available at PLoS ONE's website.

## Supporting Information

Figure S1 **LD plots for** ***IL12B*** **variants in cases.** Linkage disequilibrium (LD) plots characterizing haplotype blocks in *IL12B* in Guinea-Bissau (a, b), The Gambia (c, d), African-Americans (e, f), and Caucasians (g, h). In the first column are LD plots for pairwise D' between markers and in the second column are LD plots for pairwise r^2^ between markers. Refer to Figure 1 legend for a description of the color scheme used to define pairwise LD between polymorphisms. The haplotype blocks were created using HaploView program, version 4.1.
(PPT)

Figure S2 **LD plots for** ***IL12B*** **variants genotyped in European (CEPH) and African (Yoruba) HapMap samples.** Linkage disequilibrium (LD) plots characterizing haplotype blocks in *IL12B* in the CEPH and Yoruba populations of the HapMap. In the first column are LD plots for CEPH populations (a is the D' and c is the r^2^ LD plot) and in the second column are LD plots for the Yoruba population (b is the D' and d is the r^2^ LD plot). Please refer to Figure 1 legend for a description of the color scheme used to define pairwise LD between polymorphisms. The haplotype blocks were created using HaploView program, version 4.1.
(PPT)

Table S1 Allele and genotype frequency differences between The Gambia and Guinea Bissau.
(DOC)

Table S2 OR for significant replicated associations from Table 2 adjusted for ethnicity, age and gender.
(DOC)

Table S3 OR for African Americans unadjusted (a), OR for African Americans adjusted for age and gender (b).
(DOC)
