# Correction: Interleukin 12B (*IL12B*) Genetic Variation and Pulmonary Tuberculosis: A Study of Cohorts from The Gambia, Guinea-Bissau, United States and Argentina

**DOI:** 10.1371/annotation/468e1735-605a-40a7-a253-884a117f0e72

**Published:** 2011-02-28

**Authors:** Gerard A. J. Morris, Digna R. Velez Edwards, Philip C. Hill, Christian Wejse, Cyrille Bisseye, Rikke Olesen, Todd L. Edwards, John R. Gilbert, Jamie L. Myers, Martin E. Stryjewski, Eduardo Abbate, Rosa Estevan, Carol D. Hamilton, Alessandra Tacconelli, Giuseppe Novelli, Ercole Brunetti, Peter Aaby, Morten Sodemann, Lars Østergaard, Richard Adegbola, Scott M. Williams, William K. Scott, Giorgio Sirugo

There was an error in Table 3. P-values at column 5, line 1 (0.05), line 7 (0.05) and line 9 (0.04) should be in bold.

