# Correction: Interleukin 12B (*IL12B*) Genetic Variation and Pulmonary Tuberculosis: A Study of Cohorts from The Gambia, Guinea-Bissau, United States and Argentina

**DOI:** 10.1371/annotation/1ced138d-f6da-48a6-9db1-e1ede412a8b9

**Published:** 2011-02-28

**Authors:** Gerard A. J. Morris, Digna R. Velez Edwards, Philip C. Hill, Christian Wejse, Cyrille Bisseye, Rikke Olesen, Todd L. Edwards, John R. Gilbert, Jamie L. Myers, Martin E. Stryjewski, Eduardo Abbate, Rosa Estevan, Carol D. Hamilton, Alessandra Tacconelli, Giuseppe Novelli, Ercole Brunetti, Peter Aaby, Morten Sodemann, Lars Østergaard, Richard Adegbola, Scott M. Williams, William K. Scott, Giorgio Sirugo

There was an error in the third subheading of the Results section ("African-Americans and Caucasians"). The correct sentence is: Single locus tests of association in the African-Americans identified seven significant allelic associations at IL12B polymorphisms rs3212227 (p = 0.002), rs2421047 (p = 0.008), rs2288831 (p = 0.008), rs10631390 (p = 0.001), rs3212220 (p = 0.003), rs6894567 (p = 0.007), and rs17860508 (p = 0.002) and three significant genotypic associations at rs3212227 (p = 0.05), rs10631390 (p = 0.05) and rs6894567 (p= 0.03) (Table 2b).&rdquo;

