# Supplementary material for: Interleukin 12B (IL12B) Genetic Variation and Pulmonary Tuberculosis: A Study of Cohorts from The Gambia, Guinea-Bissau, United States and Argentina
Source: PLoS One. 2011 Feb 9;6(2):e16656. doi: 10.1371/journal.pone.0016656 (PMC3037276; doi:10.1371/journal.pone.0016656)
Supplement: Figure S1 — LD plots for IL12B variants in cases. Linkage disequilibrium (LD) plots characterizing haplotype blocks in IL12B in Guinea-Bissau (a, b), The Gambia (c, d), African-Americans (e, f), and Caucasians (g, h). In the first column are LD plots for pairwise D' between markers and in the second column are LD plots for pairwise r2 between markers. Refer to Figure 1 legend for a description of the color scheme used to define pairwise LD between polymorphisms. The haplotype blocks were created using HaploView program, version 4.1. (PPT) [file pone.0016656.s001.ppt]

## Slide 1
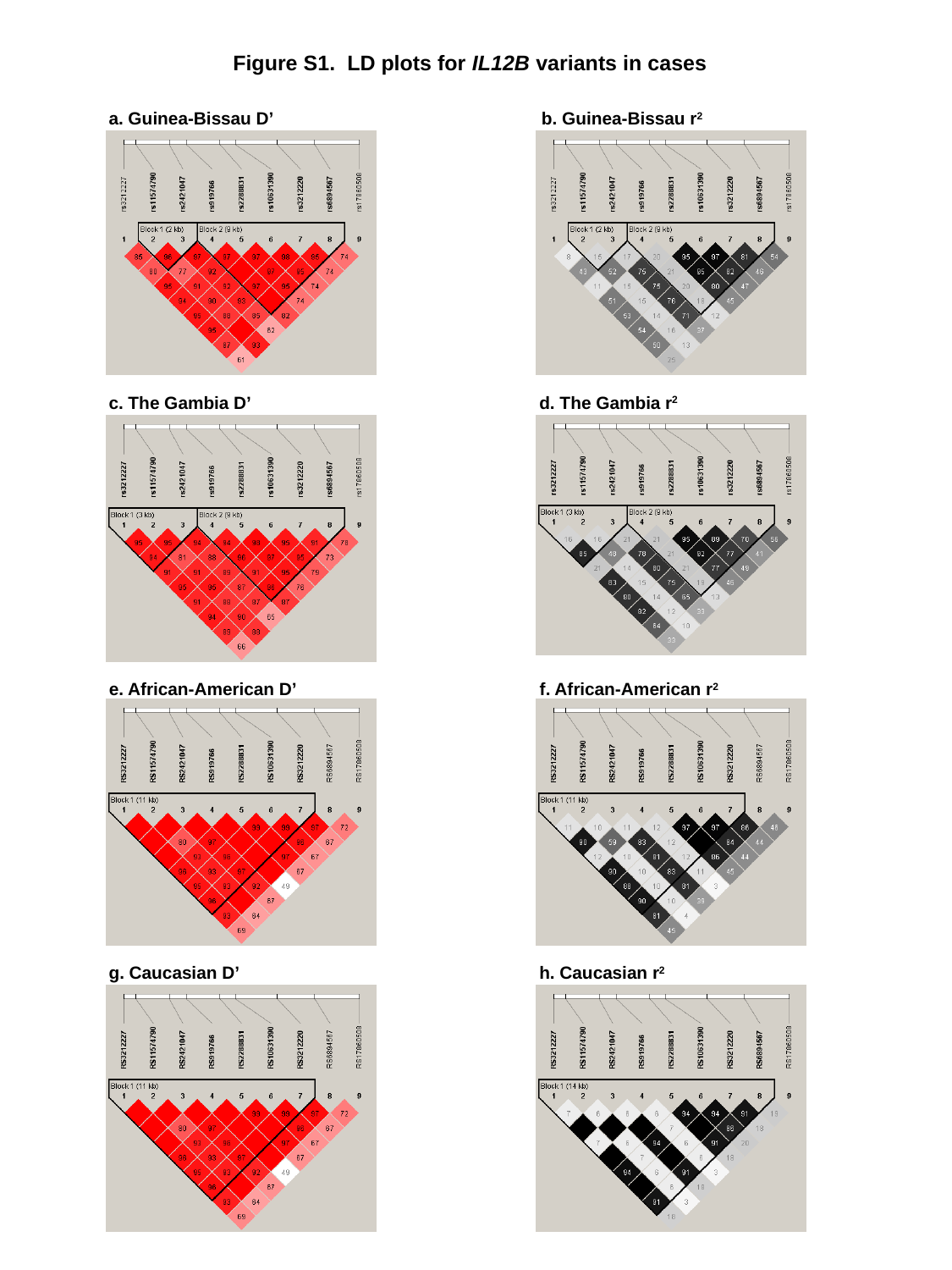

# Figure S1. LD plots for IL12B variants in cases
a. Guinea-Bissau D’
b. Guinea-Bissau r2
c. The Gambia D’
d. The Gambia r2
e. African-American D’
f. African-American r2
g. Caucasian D’
h. Caucasian r2
