# Supplementary material for: Interleukin 12B (IL12B) Genetic Variation and Pulmonary Tuberculosis: A Study of Cohorts from The Gambia, Guinea-Bissau, United States and Argentina
Source: PLoS One. 2011 Feb 9;6(2):e16656. doi: 10.1371/journal.pone.0016656 (PMC3037276; doi:10.1371/journal.pone.0016656)
Supplement: Figure S2 — LD plots for IL12B variants genotyped in European (CEPH) and African (Yoruba) HapMap samples. Linkage disequilibrium (LD) plots characterizing haplotype blocks in IL12B in the CEPH and Yoruba populations of the HapMap. In the first column are LD plots for CEPH populations (a is the D' and c is the r2 LD plot) and in the second column are LD plots for the Yoruba population (b is the D' and d is the r2 LD plot). Please refer to Figure 1 legend for a description of the color scheme used to define pairwise LD between polymorphisms. The haplotype blocks were created using HaploView program, version 4.1. (PPT) [file pone.0016656.s002.ppt]

## Slide 1
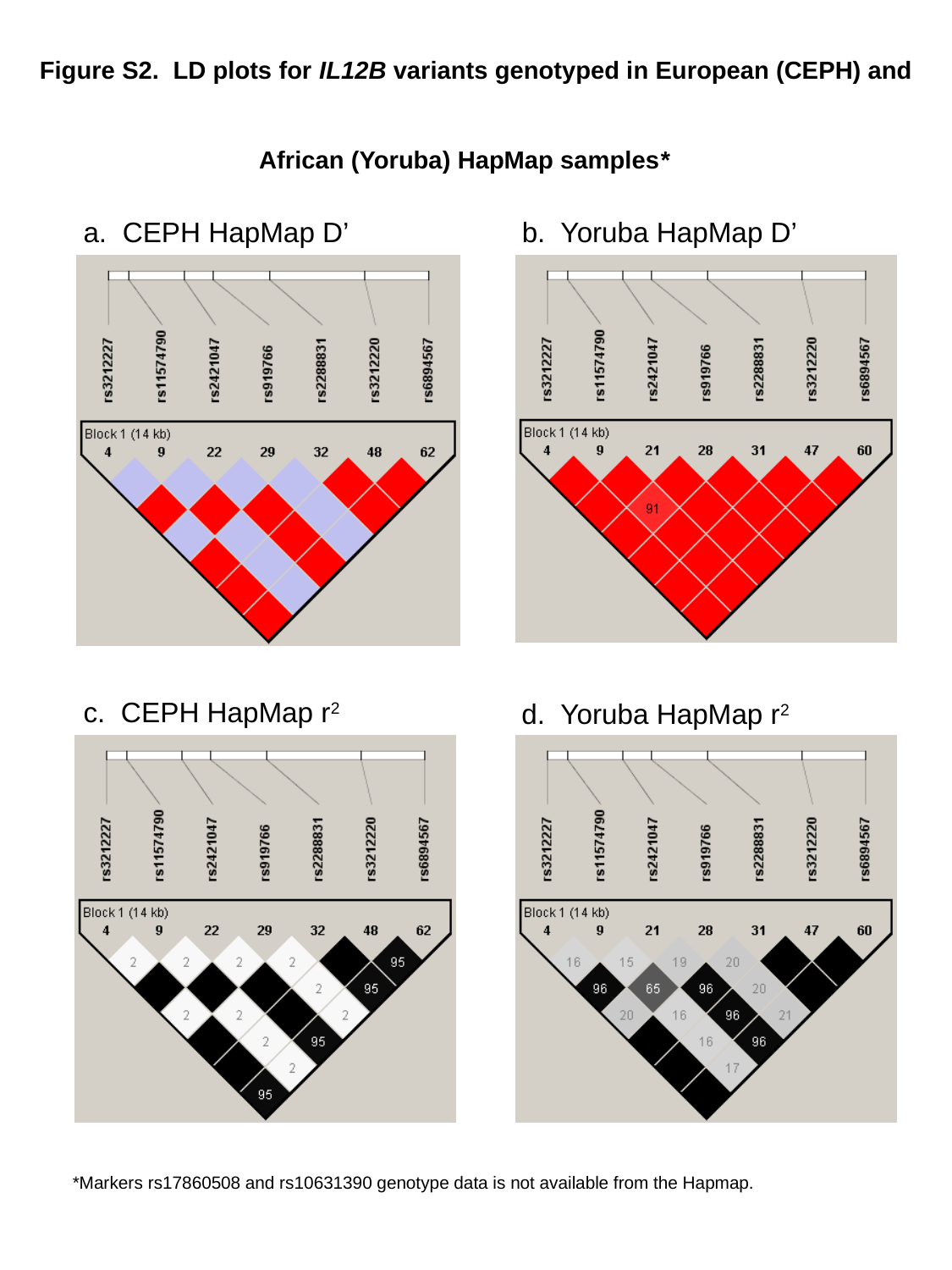

# Figure S2. LD plots for IL12B variants genotyped in European (CEPH) and African (Yoruba) HapMap samples*
a. CEPH HapMap D’
b. Yoruba HapMap D’
c. CEPH HapMap r2
d. Yoruba HapMap r2
*Markers rs17860508 and rs10631390 genotype data is not available from the Hapmap.
