# Supplementary material for: Interleukin 12B (IL12B) Genetic Variation and Pulmonary Tuberculosis: A Study of Cohorts from The Gambia, Guinea-Bissau, United States and Argentina
Source: PLoS One. 2011 Feb 9;6(2):e16656. doi: 10.1371/journal.pone.0016656 (PMC3037276; doi:10.1371/journal.pone.0016656)
Supplement: Table S1 — Allele and genotype frequency differences between The Gambia and Guinea Bissau. (DOC) [file pone.0016656.s003.doc]

**Table S1. Allele and genotype frequency differences between The Gambia and Guinea Bissau**

| ***Gene*** | **Marker** | **Allele Frequency Differences** | | **Genotype Frequency Differences** | |
| --- | --- | --- | --- | --- | --- |
|  |  | **Cases** | **Controls** | **Cases** | **Controls** |
| *IL12B* | rs3212227 | **<1.00x10^-3^** | 0.08 | **1.00x10^-6^** | 0.14 |
|  | rs11574790 | 0.50 | 0.41 | 0.78 | 0.63 |
|  | rs2421047 | 0.24 | 0.96 | 0.20 | 0.91 |
|  | rs919766 | 0.42 | 0.25 | 0.70 | 0.55 |
|  | rs2288831 | 0.80 | 0.20 | 0.89 | 0.46 |
|  | rs10631390 | 0.90 | 0.48 | 0.90 | 0.50 |
|  | rs3212220 | 0.60 | 0.95 | 0.80 | 0.84 |
|  | rs6894567 | 1.00 | 0.24 | 0.89 | 0.35 |
|  | rs17860508 | 0.55 | 0.0 | 0.77 | 0.72 |
