# Supplementary material for: Interleukin 12B (IL12B) Genetic Variation and Pulmonary Tuberculosis: A Study of Cohorts from The Gambia, Guinea-Bissau, United States and Argentina
Source: PLoS One. 2011 Feb 9;6(2):e16656. doi: 10.1371/journal.pone.0016656 (PMC3037276; doi:10.1371/journal.pone.0016656)
Supplement: Table S2 — OR for significant replicated associations from Table 2 adjusted for ethnicity, age and gender. (DOC) [file pone.0016656.s004.doc]

**Table S2. OR for significant replicated associations from Table 2 adjusted for ethnicity, age and gender**

| **Populations** | **Model (rs3212227)** | **OR** | **95% CI** | ***P*-Value** |
| --- | --- | --- | --- | --- |
| Guinea-Bissau | Additive (TT (major allele homozygote), GT, GG (minor allele homozygote)) | 0.77 | 0.59-0.99 | **0.044** |
|  | TT&GT v GG (Baseline) | 1.66 | 0.94-2.92 | 0.079 |
|  | GG&GT v TT (Baseline) | 0.79 | 0.56-1.08 | 0.142 |
| The Gambia | Additive (TT (major allele homozygote), GT, GG (minor allele homozygote)) | 1.15 | 0.88-1.51 | 0.301 |
|  | TT&GT v GG (Baseline) | 0.83 | 0.49-1.40 | 0.490 |
|  | GG&GT v TT (Baseline) | 1.23 | 0.85-1.79 | 0.279 |

* Ethnicity and gender did not show evidence for confounding (change in effect size by greater than or equal to 5%) for the association with rs3212227 in the Guinea Bissau or The Gambia populations, as is evident from the above table. Therefore we did not include an adjustment for ethnicity and gender in the results presented in the primary text of the manuscript.
