# Supplementary material for: Interleukin 12B (IL12B) Genetic Variation and Pulmonary Tuberculosis: A Study of Cohorts from The Gambia, Guinea-Bissau, United States and Argentina
Source: PLoS One. 2011 Feb 9;6(2):e16656. doi: 10.1371/journal.pone.0016656 (PMC3037276; doi:10.1371/journal.pone.0016656)
Supplement: Table S3 — OR for African Americans unadjusted (a), OR for African Americans adjusted for age and gender (b). (DOC) [file pone.0016656.s005.doc]

**Table S3. OR for African Americans unadjusted (a), OR for African Americans adjusted for age and gender (b)**

| **a** | rs# | OR | 95% CI | | *P* |
| --- | --- | --- | --- | --- | --- |
|  |  |  | Lower | Upper |  |
|  | rs3212227 | 0.78 | 0.61 | 1.00 | **0.047** |
|  | rs11574790 | 0.99 | 0.72 | 1.36 | 0.962 |
|  | rs2421047 | 0.80 | 0.62 | 1.02 | 0.073 |
|  | rs919766 | 0.96 | 0.71 | 1.30 | 0.811 |
|  | rs2288831 | 0.83 | 0.66 | 1.06 | 0.133 |
|  | rs10631390 | 0.80 | 0.63 | 1.00 | **0.050** |
|  | rs3212220 | 0.82 | 0.64 | 1.03 | 0.084 |
|  | rs6894567 | 0.76 | 0.59 | 0.98 | **0.033** |
|  | rs17860508 | 0.87 | 0.68 | 1.12 | 0.273 |
| **b** | rs# | OR^1^ | 95% CI | | *P* |
|  |  |  | Lower | Upper |  |
|  | rs3212227 | 0.87 | 0.61 | 1.24 | 0.438 |
|  | rs11574790 | 0.96 | 0.62 | 1.49 | 0.859 |
|  | rs2421047 | 0.87 | 0.60 | 1.24 | 0.437 |
|  | rs919766 | 0.84 | 0.55 | 1.29 | 0.429 |
|  | rs2288831 | 0.96 | 0.68 | 1.36 | 0.828 |
|  | rs10631390 | 0.86 | 0.62 | 1.19 | 0.368 |
|  | rs3212220 | 0.92 | 0.66 | 1.29 | 0.646 |
|  | rs6894567 | 0.84 | 0.60 | 1.19 | 0.333 |
|  | rs17860508 | 1.05 | 0.74 | 1.50 | 0.782 |

^1^ OR were calculated assuming an additive model.
